# Synthesis of a Novel Lantibiotic Using Mutacin II Biosynthesis Apparatus

**DOI:** 10.1128/spectrum.03030-22

**Published:** 2023-01-16

**Authors:** Saswati Biswas

**Affiliations:** a Department of Microbiology, Molecular Genetics, and Immunology, University of Kansas Medical Center, Kansas City, Kansas, USA; Ohio State University

**Keywords:** lantibiotics, nukacin, mutacin II, T8, oral streptococci, *S. mutans*, bacteriocins

## Abstract

Owing to extensive metagenomic studies, we now have access to numerous sequences of novel bacteriocin-like antimicrobial peptides encoded by various cultivable and noncultivable bacteria. However, relatively rarely, we even have access to these cultivable strains to examine the potency and the targets of the predicted bacteriocins. In this study, we evaluated a heterologous biosynthetic system to produce biologically active nonnative novel lantibiotics, which are modified bacteriocins. We chose Streptococcus mutans, a dental pathogen, as the host organism because it is genetically easy to manipulate and is inherently a prolific producer of various bacteriocins. We chose the S. mutans T8 strain as the host, which produces the lantibiotic mutacin II, to express 10 selected homologs of mutacin II identified from GenBank. These lantibiotic peptides either are novel or have been studied very minimally. The core regions of the selected lantibiotic peptides were fused to the leader sequence of the mutacin II peptide and integrated into the chromosome such that the core region of the native mutacin II was replaced with the new core sequences. By this approach, using the mutacin II biosynthesis machinery, we obtained one bioactive novel lantibiotic peptide with 52% different residues compared to the mutacin II core region. This unknown lantibiotic is encoded by Streptococcus agalactiae and Streptococcus ovuberis strains. Since this peptide displays some homology with nukacin ISK-1, we named it nukacin Spp. 2. This study demonstrated that the mutacin II biosynthesis machinery can be successfully used as an efficient system for the production of biologically active novel lantibiotics.

**IMPORTANCE** In this study, we report for the first time that Streptococcus mutans can be used as a host to produce various nonnative lantibiotics. We showed that in the T8 strain, we could produce bioactive lacticin 481 and nukacin ISK-1, both of which are homologs of mutacin II, using T8’s modification and secretion apparatus. Similarly, we also synthesized a novel bioactive lantibiotic, which we named nukacin Spp. 2.

## INTRODUCTION

For over 90 years, antibiotics have been used to combat bacterial infections. However, the uncontrolled use of antibiotics has led to a rapid increase in antibiotic-resistant pathogens. The emergence of multidrug-resistant bacteria and the simultaneous slowdown of research for developing new drugs in recent years pose a serious threat to human health (1, 2). Thus, there is a need for the development of novel antimicrobial agents. Antimicrobial peptides, such as lantibiotics, may serve as alternatives to traditional antimicrobial agents (3–5). Lantibiotics are ribosomally synthesized antimicrobial peptides consisting of 22 to 32 residues that are significantly modified by dedicated modification enzymes (6–8). Generally, the structural genes of the lantibiotics and the genes required for synthesis, modification, secretion, and immunity are organized in an operon or are located in an adjacent region (7, 8). The lantibiotic peptides are first synthesized as prepeptides with specific leader sequences that are recognized by the cognate modification enzymes for the sequence-specific dehydration and circularization of the core peptide (9, 10). The leader sequence also provides specificity for the exporter proteins and is ultimately cleaved off to generate mature lantibiotics (10–12). Often, a standalone regulator or a two-component system comprising a histidine kinase and a response regulator is also included within the lantibiotic operon or the nearby region (13, 14). Lantibiotics are able to form pores, inhibit peptidoglycan synthesis, and cause ATP depletion in target cells (15). The diverse modes of action of lantibiotics have generated significant research interest in using them either as standalone therapeutic agents or to potentiate the action of existing antibiotics (5, 16, 17). The most widely used lantibiotic is nisin, which has been used worldwide as a food preservative for decades (18–20). The use of lantibiotics in medicine is still limited, although several candidates are in clinical and preclinical trials (21, 22).

With the advent of genomics and metagenomics data, an abundance of novel lantibiotic sequences are now publicly available. However, the main challenge remains the procurement of the original producer strains, which is necessary to characterize the lantibiotics and identify the target bacteria. Furthermore, some lantibiotic gene clusters are often not expressed in the native organism under laboratory conditions (23). Thus, determining the optimal culture conditions can also be a daunting task. To overcome these obstacles, an alternative approach has recently been explored where nonnative lantibiotics are expressed in a heterologous host whose culture conditions have been established. However, the genome sequencing data available in GenBank, including metagenomic data, often have incomplete information on the predicted lantibiotic’s biosynthetic gene cluster, which is often very large (7). On the other hand, the structural genes encoding lantibiotics are only a few hundred bases. Thus, the expression and production of such lantibiotics using the heterologous biosynthetic gene cluster of the host are an attractive alternative that needs to be further explored.

Streptococcus mutans is an oral pathogen that causes dental caries (24). For successful colonization, this organism displaces commensal bacteria that are already present on the tooth surfaces as a protective layer. One of the arsenals that S. mutans uses is the secretion of antimicrobial peptides to inhibit competing bacteria. This is one of the reasons why it is naturally a prolific producer of antimicrobial peptides, including lantibiotics (25). Because of this trait, S. mutans can be used as a host to express various lantibiotic and nonlantibiotic peptides. S. mutans is known to secrete various single-peptide lantibiotics as well as two-peptide lantibiotics. Among the single-peptide lantibiotics, mutacin I, mutacin II, mutacin III, and K8 are well studied, and SmbAB is the only two-peptide lantibiotic produced by S. mutans (25, 26). Thus, this organism offers a variety of native lantibiotic sequence templates to explore and identify novel lantibiotics from GenBank. Other applicable qualities of this organism are that it is naturally competent and genetically very easy to manipulate; furthermore, many strains are classified as biosafety level 1 (BSL-1) organisms (27).

In this study, we used the S. mutans T8 strain as the host; it primarily secretes a single-peptide lantibiotic, mutacin II, which is classified as a type A-II lantibiotic (14, 28). Lantibiotics are classified as type A-I and type A-II based on the nature and mechanisms of modification of the lantibiotic peptides (29). Type A-II lantibiotics use the simplest modification machinery and require only a single LanM-type protein containing two domains with enzymatic activities of both dehydration and cyclization (30). Some well-known type A-II lantibiotics include lacticin 481 (L-481), mutacin II, nukacin ISK-1, bovicin HJ50, salivaricin A, and salivaricin B; therefore, they comprise one of the largest groups of lantibiotics (6, 29). On the other hand, type A-I lantibiotics use two separate enzymes, LanB for dehydration/modification and LanC for cyclization (31). The most well-studied lantibiotic, nisin, as well as mutacin 1140 belong to this category (32). Another characteristic of the majority of type A-II lantibiotics is that the exporter enzyme complex encodes the leader peptide processing domain along with the ABC transporter function. In contrast, both nisin and mutacin 1140 use a separate peptidase for processing the leader peptide (32). In this study, we selected 10 promising mutacin II homologs from a list of 30 that are either novel or less well explored. We expressed these homologs in the S. mutans T8 strain to establish a heterologous system and identify novel activity. We were able to successfully obtain a novel lantibiotic that has inhibitory activity against pathogens such as Listeria monocytogenes.

## RESULTS

### Overall strategy used to establish a heterologous platform for evaluating novel lantibiotics.

**FIG 1:**
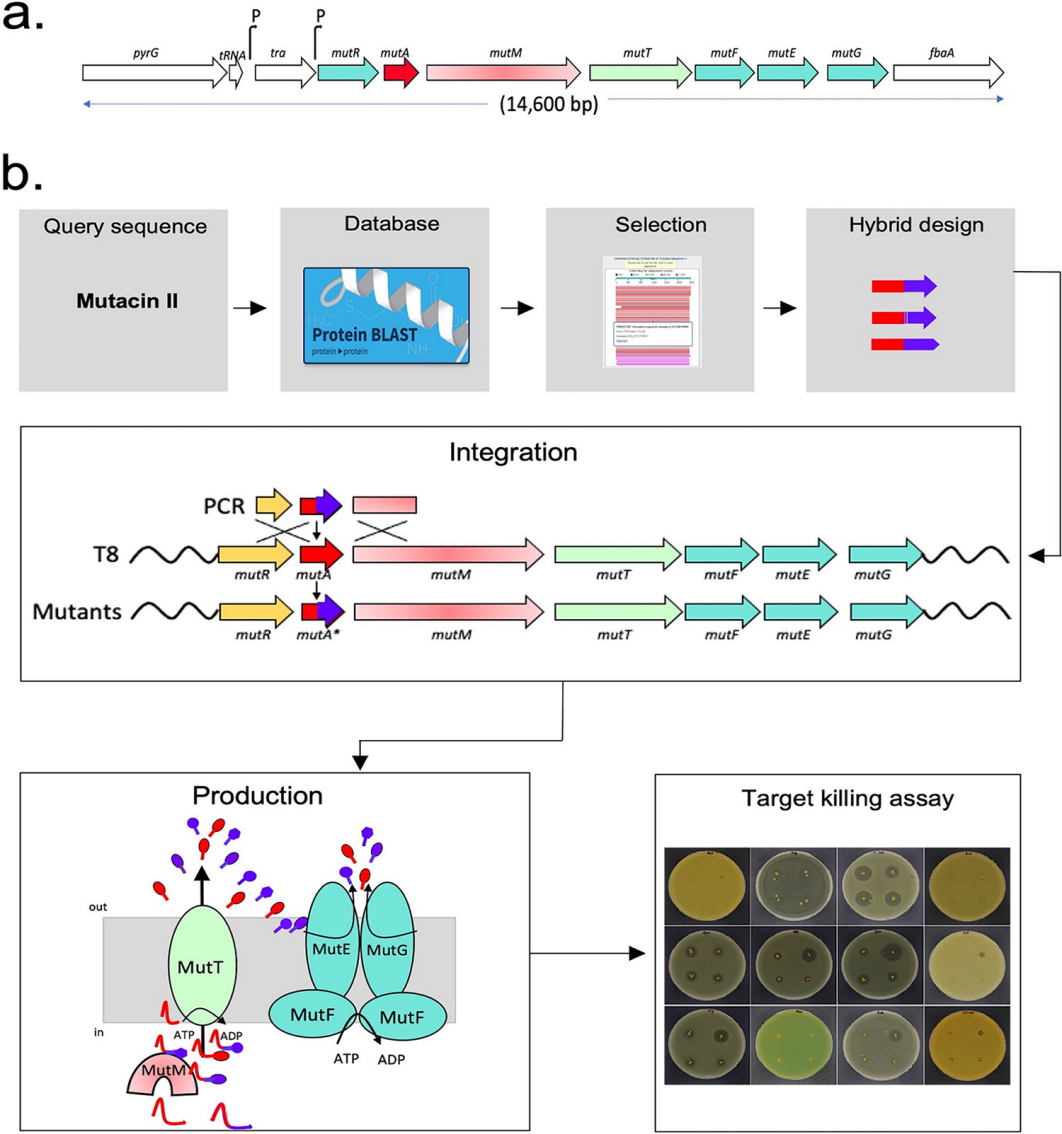
General overview of novel lantibiotic production by accessing solely the lantibiotic sequences from GenBank. (a) Genomic organization of the mutacin II locus. The first gene, *mutR*, encodes a putative transcription factor of the helix-turn-helix XRE superfamily. The second gene, *mutA*, is the structural gene of mutacin II. The genes *mutM* and *mutT* encode the modification enzyme and the transporter, respectively. The ABC transporter complex genes *mutF*, *mutE*, and *mutG* constitute the immunity protein complex. The bent arrow indicates the putative promoter sequence. The putative transposase *tra* is also a part of this locus. The locus is integrated between the conserved leucine tRNA and the *fbaA* genes. (b) Publicly available data from GenBank were used to select the core sequences of the mutacin II homologs. New core peptides were integrated by replacing the core of mutacin II in the T8 chromosome by generating a hybrid of the native leader and the core from the homologs. The entire biosynthetic machinery of mutacin II, including the modification enzyme and the exporter and immunity proteins, was used to generate novel and bioactive lantibiotics. Deferred-antagonism assays were carried out with several target strains for the assessment of activity.

The locus that has the genes for mutacin II biosynthesis and the gene organization are shown in Fig. 1a (14, 33). The operon includes a total of seven genes, the first of which, *mutR*, encodes a transcriptional regulator. The next gene in the operon, *mutA*, is the structural gene for the mutacin II lantibiotic. *mutA* encodes two distinct regions: a leader peptide of 26 amino acids and a core region of 27 residues. The leader peptide of MutA is required for the specificity of the modification enzyme LanM, which is 901 residues long and contains both dehydratase and cyclase activities. The leader peptide is also recognized by the exporter protein MutT, which is 705 residues long. The last set of three genes, *mutFEG*, encodes the cognate immunity function. The transposase (*tra*) gene is thought to be involved in the integration of the mutacin II biosynthetic locus at the site located on the T8 chromosome that is flanked by the tRNA(Leu) and *fbaA* (fructose-1,6-bisphosphate aldolase) genes (33).

The strategy for evaluating homologs in the heterologous host T8 is depicted in Fig. 1b. We first used the mutacin II prepeptide sequence (MutA) as the query for a BLASTP search to identify homologs of MutA. We selected homologs that were either not characterized or characterized very little. We then applied a fusion PCR method to generate hybrids where the leader peptide of mutacin II is fused to the core peptide of the putative lantibiotics. Using the ~300-bp regions of homology up- and downstream of the *mutA* gene, the hybrid PCR products were integrated into the T8 chromosome by replacing the native core of the *mutA* gene. Next, the strains were checked for accuracy by sequencing. Our assumption is that the native modification enzyme MutM will modify the nonnative cores since the leader peptide is not altered. Similarly, the transporter MutT will be able to recognize the native leader peptide and export the mature lantibiotic after cleavage at the proper site. Finally, the modified strains were tested against a panel of 12 target pathogens, including a few from the ESKAPE (Enterococcus faecium, Staphylococcus aureus, Klebsiella pneumoniae, Acinetobacter baumannii, Pseudomonas aeruginosa, and Enterobacter species) category (34).

**FIG 2:**
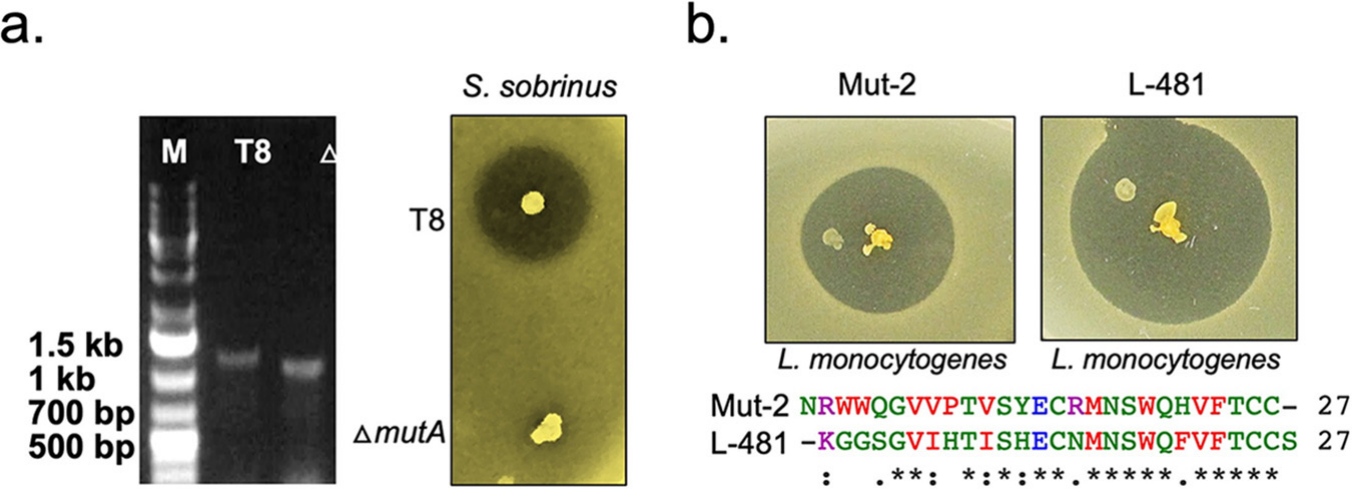
Evaluation of the promiscuity of the biosynthetic apparatus of mutacin II. (a) A clean knockout strain, Δ*mutA*, was constructed, and the size was verified by agarose gel electrophoresis. For phenotypic assays, bacterial cultures were stabbed onto THY agar plates and incubated overnight at 37°C under microaerophilic conditions. The plates were then overlaid with soft agar containing the *S. sobrinus* indicator strain for mutacin II. The zones of inhibition (ZOIs) of the indicator strains were observed after incubation overnight. The observation is based on the results of four separate experiments, and a representative plate is shown. (b) The mutacin II synthesis machinery is promiscuous. The homology of mutacin II and lacticin 481 is not extensive. Still, L-481 produced using the mutacin II machinery is active and produces a clear zone against *Listeria*.

Since S. mutans T8 harbors various bacteriocin loci, we wanted to confirm that under the experimental conditions used for this study, the mutacin II gene cluster is actively expressed in this strain. As shown in Fig. 2, we observed that the T8 strain produces a distinct zone of inhibition (ZOI) with a diameter of ~15 mm against the Streptococcus sobrinus strain, which traditionally serves as an indicator strain for mutacin II (31). To further confirm that the observed ZOI was due to mutacin II and not other bacteriocins, we generated a clean *mutA* knockout strain (Δ*mutA*) and tested it against S. sobrinus. As shown in Fig. 2a, the deletion of *mutA* completely abolished mutacin production since we observed no ZOI against *S. sobrinus*. However, when the Δ*mutA* strain was tested against 11 other target bacteria, the ZOI was negligible, except for Streptococcus constellatus and Enterococcus faecium (and also Enterococcus faecalis [see Fig. S3 in the supplemental material]). The ZOI produced against them was consistently larger than that against the wild-type T8 strain. Apart from this observation, overall, S. mutans T8 can be used as a desired heterologous host for lantibiotic expression since it can produce copious amounts of mutacin II under the laboratory conditions used in this study (Fig. 2a).

### Evaluating the promiscuity of the mutacin II biosynthetic machinery.

We first expressed the lacticin 481 core peptide from the mutacin II locus. We chose lacticin 481 since it belongs to the same class, and its target is known. We observed that the active product was produced and secreted from T8 by visualizing target inhibition on an agar plate (Fig. 2b). Since the sequence similarity between mutacin II and lacticin 481 is not extensive (Fig. 2b), we concluded that the modification enzyme MutM and the immunity complex MutFEG of mutacin II potentially could display broad specificity (promiscuity) to accommodate variant production.

### Evaluating novel homologs using the mutacin II biosynthetic machinery for bioactivity.

As indicated above, we used mutacin II with the leader peptide sequence for a BLASTP search and shortlisted the top 30 closest homologs (Table S1). We then selected only 10 for further study by focusing on their novelty and sequence variation from each other. Additionally, we limited the selection to only class II lantibiotics, most of which are encoded by streptococci. The core peptide sequences of the selected homologs and the gene identifications used for subsequent analyses are listed in Table 1. Among the novel homologs, five were monopeptides and five were members of multipeptide clusters (Table 1 and Fig. S1).

**TABLE 1:** List of tested mutacin II homologs^*a*^

Locus	Core sequence	Size (no. of residues)	Organism	GenBank accession no.
WT*	NRWWQGVVP**T**V**S**YECRMN**S**WQHVF**T**CC	27	Streptococcus mutans	WP_080035512.1
1*	KGKG**S**GVIK**T**L**T**HECKMN**T**YQAIL**T**CC	27	*Weissella cryptocerci*	WP_133363361.1
2	AGHGVN**T**I**S**AECRWN**S**LQAIF**T**CC	24	Streptococcus equi 2	WP_080004879.1
3*	GDGVIK**T**I**S**HECAMN**T**WQFIF**T**CC**S**	25	*Clostridium indicum*	WP_117418566.1
4	GKNGAIK**T**I**S**HECHMN**S**WQFLF**T**CC**S**	26	Streptococcus equi 1	WP_037580734.1
5	GKNGVFK**T**I**S**HECHLN**T**WAFLA**T**CC**S**	26	Streptococcus pyogenes	WP_136018286.1
6	GKNGVFK**T**I**S**HECHMN**S**WQFLF**T**CC**S**	26	Streptococcus suis	WP_002935049.1
7	RKNGVFK**T**I**S**HECHLN**T**WAFLA**T**CC**S**	26	Streptococcus macedonicus	WP_093528619.1
8*	**S**RFWQGVVP**T**V**S**YECRMN**S**WQ**S**IF**T**CC	27	Streptococcus gallolyticus 1	WP_074596227.1
9*	**S**RWWQGVVP**T**V**S**HECNMN**S**FQHVF**T**CC	27	Streptococcus sp. 1	WP_080567926.1
10*	KKKGNDGAIP**T**I**S**HDCHMN**S**WQFIF**T**CC**S**	29	Streptococcus sp. 2	WP_079260956.1

a The conserved cysteines are underlined, and the serine and threonine residues are indicated in boldface type. Core sequences from the monopeptide-encoding loci (1, 3, 8, 9, and 10 [indicated by *]) and core sequences from the multiple-lantibiotic gene cluster loci (2, 4, 5, 6, and 7) are shown. We considered locus 9 as a monopeptide, but no locus information is available except for the lantibiotic sequence. WT, wild type.

**FIG 3:**
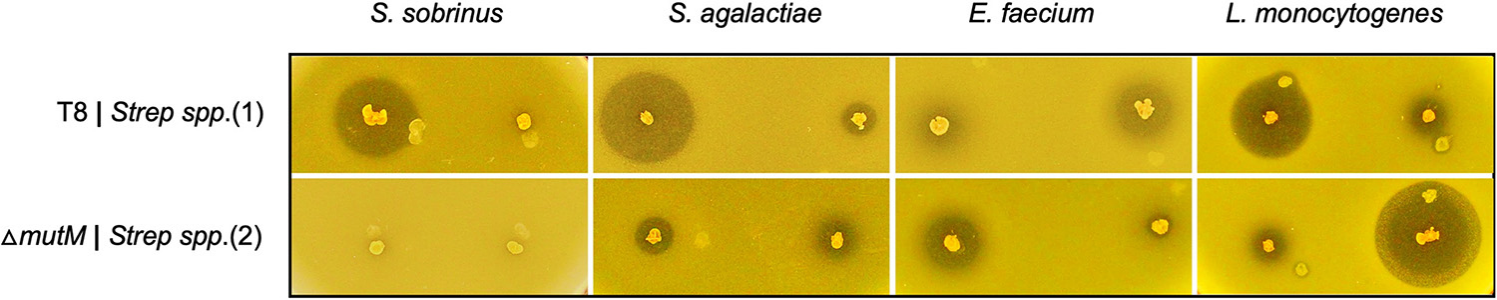
Antimicrobial activity of the novel lantibiotic homolog from Streptococcus spp. 2. The results of a deferred-antagonism assay are shown, with T8 and the Δ*mutA* mutant as positive and negative controls, respectively, and two homologs of mutacin II from Streptococcus spp. 1 and Streptococcus spp. 2 were expressed in T8 as samples. Four out of 12 indicator strains that were used to measure inhibition are shown. Experiments were repeated at least three times, and a representative set is shown. We observed the halo produced by a novel lantibiotic from Streptococcus spp. 2 against the pathogen L. monocytogenes.

After integrating the selected lantibiotic-encoding genes into the mutacin II locus, each strain was then evaluated for inhibitory activity against a panel of 12 pathogenic bacteria (Table 2). We used T8 and the Δ*mutA* mutant as positive and negative controls, respectively. Out of the 10 candidate homologs tested, we found one novel lantibiotic encoded by multiple streptococcal strains, Streptococcus sp. 2 that showed inhibitory activity against the Gram-positive pathogen L. monocytogenes (Fig. 3 and Table 2). The spectrum of activity of this new lantibiotic was different from that of the wild-type T8 strain, as L. monocytogenes was the only strain that was sensitive. For the remaining lantibiotics tested, we observed that they all displayed ZOIs similar to that of the Δ*mutA* strain (Table 2).

**TABLE 2:** Comparison of the antimicrobial activities of mutacin II homologs^*a*^

Homolog	Target											
	A. baumannii	Streptococcus anginosus	*S. constellatus*	Enterobacter aerogenes	E. faecium	S. agalactiae	Streptococcus gordonii	Streptococcus iniae	L. monocytogenes	P. aeruginosa	*S. sobrinus*	S. aureus
T8	*−*	++++	++	−	+/−	++++	+++++	+	*++++*	*−*	+++	+
Δ*mutA*	−	+	+++(+)	−	++	+	++(+)	−	+	−	−	−
S. equi 1	−	+	++	−	+	+	++	−	+	−	−	−
S. equi 2	−	+	++	−	+	+	++	−	+	−	−	−
Streptococcus spp. 1	−	+	++	−	+	+	++	−	+	−	−	−
Streptococcus spp. 2	−	−	++	−	−	+	+	−	++++++	−	−	−
S. pyogenes	−	+	++	−	+	+	++	−	+	−	−	−
S. suis	−	+	++	−	+	+	++	−	+	−	−	−
*W. cryptocerci*	−	+	++	−	+	+	++	−	+	−	−	−
*S. macedonicus*	−	+	++	−	+	+	++	−	+	−	−	−
*C. indicum*	−	+	++	−	+	+	++	−	+	−	−	−
S. gallolyticus	−	+	++	−	+	+	++	−	+	−	−	−

a The zones of clearing are represented relative to the smallest halo, indicated as “+,” and no halo is represented by “−.” The assay was repeated two times.

### Genomic arrangement of the novel lantibiotic locus and its homologs.

**FIG 4:**
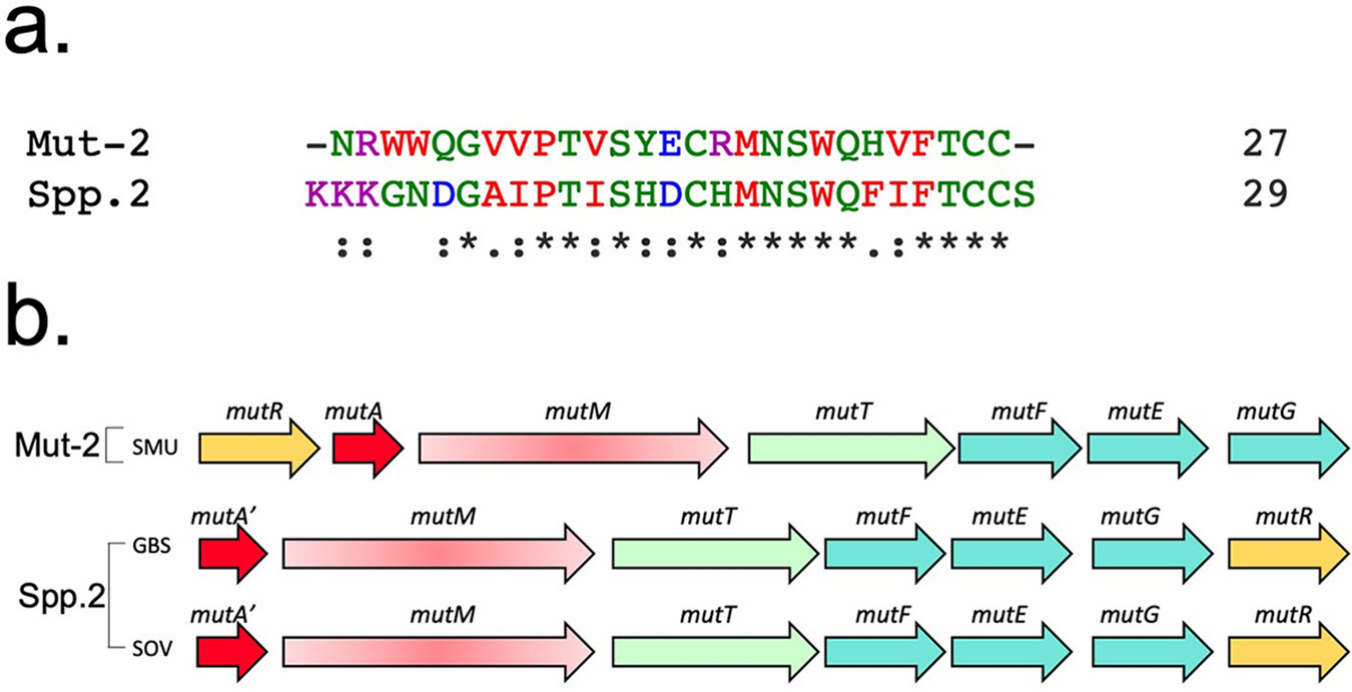
Comparison of the novel lantibiotic Spp. 2, a homolog from Streptococcus spp. 2, to mutacin II. (a) Sequence alignment of mutacin II (Mut-2) and its homolog, the novel mutacin Spp.2. Sequences were aligned using ClustalW. (b) The genomic locus from the original source of the novel lantibiotic Spp.2 compared with that of the biosynthetic gene cluster of mutacin II. The source strains are S. mutans T8 (SMU) (GenBank accession number CP044492.1), S. agalactiae LMG 14609 (GBS [group B streptococcus]), and *S. ovuberis* CCUG 69612 (SOV).

The GenBank accession number for the lantibiotic that generated the active molecule is WP_079260956.1, which is referred to as Streptococcus spp. 2. A further BLASTP search with the core peptide identified two streptococcal strains, S. agalactiae LMG 14609 and Streptococcus ovuberis CCUG 69612, that naturally encode the peptide. The whole-genome (shotgun) sequences for these two strains are also available, and this lantibiotic has not been previously characterized. Upon closer analysis, we observed that this lantibiotic has relatively less primary sequence homology with mutacin II, in which nearly 52% of the residues in the core sequence are nonidentical (Fig. 4a). However, the genomic organization of the putative biosynthesis operon is very similar to that of the mutacin II locus, with the exception that the regulator is the last gene of the operon instead of the first gene of the lantibiotic gene cluster (Fig. 4b).

**FIG 5:**
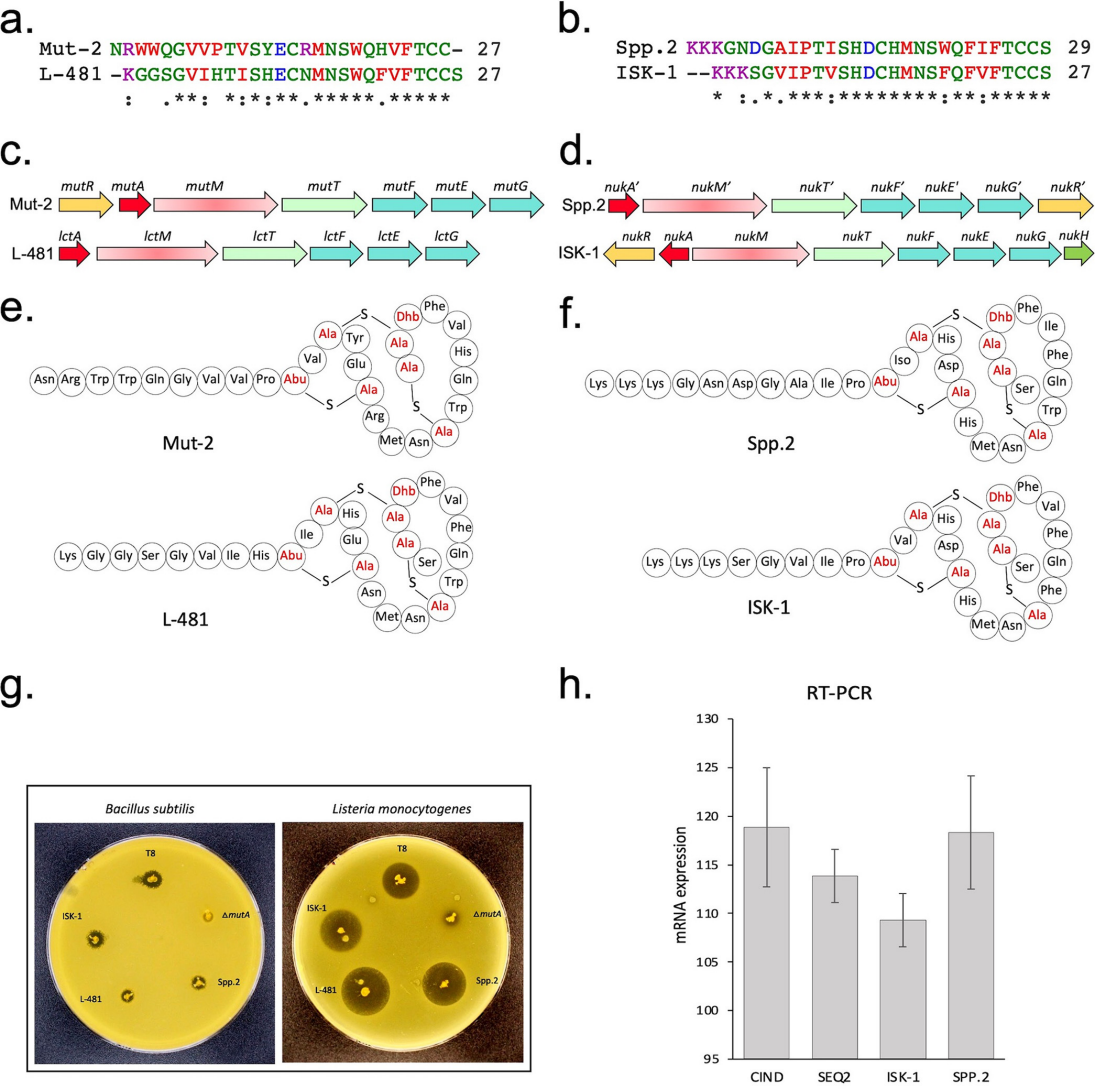
Testing the expression of nukacin ISK-1, an Spp. 2 homolog, in T8 and comparison of all of the active products. (a) Alignment of mutacin II (Mut-2) with its homolog lacticin 481 (L-481). (b) Alignment of nukacin Spp. 2 with its homolog nukacin ISK-1. (c) Comparison of the biosynthetic loci of mutacin II and lacticin 481. (*d*) Comparison of the biosynthetic loci of nukacin Spp. 2 and nukacin ISK-1. (e) Structure of the lantibiotics mutacin II and lacticin 481. (f) Predicted putative structure of the lantibiotic nukacin Spp. 2 based on homology with nukacin ISK-1. (g) Deferred-antagonism assays were performed as described in the legend of Fig. 2a. Besides the Δ*mutA* strain, T8 and the homologs Spp. 2, L-481, and ISK-1 expressed in T8 produced halos against the B. subtilis and L. monocytogenes indicator strains. Observations were repeated at least three times, and representative plates are shown. (h) The expression levels of the homologs from *C. indicum* (CIND), S. equi 2 (SEQ2), *S. warneri*, and Streptococcus spp. 2 expressed in T8 were measured by RT-PCR.

The genomic arrangement of the lantibiotic locus of lacticin 481, a close homolog of mutacin II, is also very similar (Fig. 5a and c) (6). However, we found that the novel lantibiotic showed high primary sequence homology with the lantibiotic nukacin ISK-1 instead of mutacin II (35). This novel lantibiotic contains only 26% nonidentical residues compared to nukacin ISK-1. Moreover, both have a triple-lysine signature motif present at the *N*-terminal end. Therefore, we have named the novel lantibiotic nukacin Spp. 2 (Fig. 5b). We also compared the locus organization of nukacin Spp. 2 with the locus organization of nukacin ISK-1 and observed a few variations (Fig. 5d). In the nukacin ISK-1 locus, the regulator is at the beginning of the locus, and the regulator and the structural gene of nukacin ISK-1 are expressed in the opposite direction relative to the rest of the locus. Furthermore, in the nukacin ISK-1 locus, an additional gene, *nukH*, is present at the end, which might encode an immunity function (36).

### Evaluating the heterologous system for the expression of a nukacin Spp. 2 homolog.

Both lacticin 481 and nukacin ISK-1 are lantibiotics that have been relatively well studied for their biological activities (35). While they both display some sequence similarity with mutacin II and nukacin Spp. 2, respectively (Fig. 5a and b), the sequence conservation between lacticin 481 and nukacin ISK-1 is limited. To test whether this heterologous system with the MutM modification enzyme can be applied to produce this biologically active peptide, we used an approach similar to the one described above and inserted the core peptide region with the leader peptide of mutacin II. We then screened the hybrid peptides secreting S. mutans T8 against Bacillus subtilis and L. monocytogenes for inhibitory activity. As shown in Fig. 5g, we observed that the heterologously expressed nukacin ISK-1 peptide was able to inhibit these two indicator strains. Taken together, these results indicate that the mutacin II biosynthetic apparatus can be used to produce diverse bioactive lantibiotics.

### Nonbioactive lantibiotic homologs are transcriptionally expressed.

We observed that the majority of the mutacin II homologs that we tested were biologically inactive (Table 2). Since the expression of lantibiotic loci is tightly regulated and often depends on the immunity function of the biosynthesis apparatus (25, 37), it is possible that these nonactive hybrid lantibiotics are not expressed in S. mutans T8. To confirm that their expression levels are comparable, we randomly selected one nonactive homolog (CIND) from the monopeptide group and one nonactive homolog (SEQ2) from the multipeptide cluster group for transcriptional analysis by a semiquantitative reverse transcription PCR (RT-PCR) assay. For comparison, we used the hybrid-, nukacin Spp. 2-, and nukacin ISK-1-expressing strains that produced the bioactive lantibiotics. The RT-PCR data suggest that all of the hybrid lantibiotics were expressed in S. mutans T8 (Fig. 5h, Fig. S1, and data not shown). However, we noticed that the expression levels of all of the constructs were not at the same degree. Taken together, these data suggest that some lantibiotic homologs are transcriptionally expressed well and that some were not expressed in the S. mutans host.

## DISCUSSION

Numerous lantibiotic sequences are now available in GenBank, but only about 100 have been biologically characterized to date (38). In this study, we established a heterologous system where various lantibiotic-encoding genes can be expressed in S. mutans using the host’s native lantibiotic biosynthetic machinery. The only prerequisite is the *in silico* access of the prepeptide sequences (47 to 55 residues) (6) so that they can be integrated into the chromosome of the host. We chose S. mutans as the production host because this species is known to produce large quantities of diverse lantibiotics with a broad range of target bacteria (26). Moreover, in general, S. mutans is naturally transformable; therefore, genetic manipulation is easily feasible (39). Among others, the main advantage of this strategy is that one does not need to acquire the original lantibiotic-producing strains, and also, there is no need for the laborious optimization of the growth conditions of the bacteriocin producer strains. This strategy is also applicable to nonculturable bacteria as well as bacteria whose complete genome sequences are unavailable. Recently, several strategies have been used where either the entire biosynthetic operon or the majority of the genes were transferred in a heterologous strain background to produce bioactive lantibiotics, but only limited studies have used only the structural genes coupled with the entire biosynthesis apparatus of another lantibiotic (38). This study is another one where we used the entire biosynthetic apparatus of mutacin II for the production of nonnative lantibiotics.

With this approach, we tested only 10 curated putative lantibiotics. The sequences that we selected, although extracted from GenBank based on mutacin II homology, are diverse compared to each other (Table 1). However, we were successful in generating one bioactive lantibiotic, nukacin Spp. 2, which showed strong inhibitory activity against L. monocytogenes. Thus, this approach is highly effective and comparable to that in a previous study where the authors used a promiscuous nisin biosynthesis apparatus to obtain 5 bioactive nisin-like lantibiotics from 55 tested sequences belonging to ~20 different genera (38). We observed that nukacin Spp. 2 is specifically active against listeria and not the other bacteria that we tested. On the other hand, mutacin II is active against 6 different bacteria among the 12 targets. Thus, the inhibitory activity of nukacin Spp. 2 is relatively specific. However, nukacin Spp. 2 is more active than mutacin II (ZOI diameter of 15 mm versus 10 mm) (Fig. 3). Nukacin Spp. 2 is also active against B. subtilis, albeit the activity is low compared to the activity against listeria. Nevertheless, the activity was similar to those of mutacin II and nukacin ISK-1 (Fig. 5). Of note, nukacin Spp. 2 is naturally encoded by S. agalactiae and *S. ovuberis*; they are both pathogenic species (40, 41), while S. mutans is classified as a BSL-1 organism according to the ATCC. Thus, from a production point of view, it is better to use S. mutans than the pathogenic natural producers.

Strangely, the primary sequence of nukacin Spp. 2 is not the most identical to the mutacin II core sequence, but it has the highest identity to nukacin ISK-1, originally isolated from Staphylococcus warneri (42), despite the fact that the native producers of nukacin Spp. 2 are S. agalactiae and *S. ovuberis*. Nevertheless, the native locus organization of the nukacin Spp. 2 biosynthetic gene cluster is highly similar to that of the mutacin II locus except that the regulator is the last gene instead of the first gene (Fig. 4). In contrast, the nukacin ISK-1 locus is somewhat different from the nukacin Spp. 2 locus; the principal difference in the nukacin ISK-1 locus is that the lantibiotic and the regulator are expressed in the opposite direction relative to the rest of the genes of the lantibiotic’s biosynthesis gene cluster.

Both mutacin II and nukacin ISK-1 belong to the lacticin 481 group of lantibiotics (6). Lantibiotics from this group are highly diverse at the primary sequence level but are structurally conserved (6, 10, 43). Furthermore, lantibiotics belonging to the lacticin 481 group have a broad target range (6). When we examined both lacticin 481 and nukacin ISK-1 for modification and secretion by the mutacin II biosynthetic machinery, we found that these two lantibiotics were bioactive (Fig. 5g). This observation suggests that, indeed, the mutacin II biosynthetic machinery can be successfully utilized to produce various lantibiotics or at least those that belong to the lacticin 481 group.

We were unable to observe any bioactivity of the 9 lantibiotic peptides from the list of 10 when expressed in S. mutans T8. This could be due to several reasons. First, it is possible that the modification enzyme MutM was unable to recognize or fully modify the lantibiotic peptides to make them bioactive. MutM belongs to the LanM family of lanthipeptide synthetases, and they are further divided into different groups (10). While some of the LanM family enzymes are promiscuous, such as ProcM, many others are not (10, 44). MutM may belong to the latter category, like LctM, which modifies lacticin 481 (45). However, if an active product is generated, we can assume that intramolecular bonds are formed based on mutagenesis studies of mutacin II that showed that all internal bond formations are necessary for the activity (46). Second, the leader peptide processing site might not be accurately mapped; therefore, the transporter enzymes may have failed to process the prepeptide during secretion or may have secreted an inactive product. A study of mutacin II showed that the deletion of the first asparagine residue caused a loss of activity due to improper processing (46). While designing the core peptides, we determined the leader processing site after the canonical double-glycine motif GG, GA, or GS based on the alignment (Table 1; see also Table S1 in the supplemental material) (46). However, cleavage can also occur after the AG motif (29). In that case, the first residues of the cores from Streptococcus equi 1 (strain 1), S. equi 2 (strain 2), S. pyogenes, and S. suis would be different (Table 1 and Table S1). Thus, not having the correct first residue after the leader cleavage site might be responsible for the diminished activity of the hybrids. Along the same line, there are three consecutive G’s present at the cleavage site in Clostridium indicum, and the double-glycine motif included the first two G’s, leaving the third glycine as the first residue of the core peptide. Third, the immunity protein complex MutFEG was unable to provide protection against the new lantibiotics, which prompted the cells to suppress the secretion of the newly synthesized variants. In this case, membrane stress is sensed in the producer organism by a two-component signaling system such as LiaRS, and the producer organism is able to shut down the synthesis of the lantibiotics (25, 37, 47). We tested the expression of mutacin 1140, a type A-I lantibiotic that requires two separate modification enzymes for dehydration and cyclization; however, the immunity protein complex is of the MutFEG type (48, 49). When we tested its production using the mutacin II biosynthesis apparatus, we were unable to observe any bioactive molecule against the known target Staphylococcus aureus (Fig. S2d). We also measured transcription and found that the hybrid mutacin 1140 is expressed at a very low level in the T8 strain (Fig. S2e). Thus, it is possible that mutacin 1140 was, to some extent, modified by the MutM enzyme, but the immunity complex might not have provided protection against this structurally different lantibiotic. As a result, the expression of this hybrid was suppressed by a feedback mechanism.

However, in a previous study, we showed that the immunity protein SmbFT of a two-peptide mutacin, SmbAB, can also recognize two other closely related two-peptide lantibiotics, haloduracin and gallolacticin, to provide protection (25). Furthermore, the MutFEG-type immunity complex is also known to provide protection broadly (50). Thus, immunity function might not be an issue for our system of expressing the type A-II class of lantibiotics. Finally, although we used a diverse target species to screen for bioactivity, the total number of strains used in the array was limited (12 strains), which raises the possibility that the array might have excluded the actual target organisms.

It is important to mention that for some of the lantibiotics that did not show any bioactivity, we noticed from the locus information for the strains that many of the loci harbor more than one type A lantibiotic structural gene. The exceptions were Weissella cryptocerci, which encoded the lantibiotic under GenBank accession number WP_133363361, or Streptococcus spp. 2, which encoded nukacin Spp. 2 (Table 1). Thus, it is possible that more than one lantibiotic structural gene might be necessary for bioactivity (similar to two-peptide lantibiotics such as SmbAB [25, 51]). However, lantibiotic locus information for streptococcal strains Streptococcus spp. 1 is not available in GenBank. This represents a scenario where the current strategy can still use the short lantibiotic sequence for evaluating its activity in the absence of complete information on the entire gene cluster, including the modification and secretion machinery. If the lantibiotic sequence is accessed from metagenomic data, which are often incomplete, this strategy might be the only avenue to explore promising novel lantibiotics. Therefore, more research should be done to explore such heterologous systems.

The T8 strain primarily secretes mutacin II, although it encodes several other bacteriocin-like peptides (14, 15, 33, 46). We found that the deletion of the structural gene *mutA* abolished mutacin II production against the *S. sobrinus* indicator strain (Fig. 2a). Interestingly, we also observed that the deletion of *mutA* also led to increased inhibition of E. faecium (Fig. 3). While the exact reason for this increased sensitivity to E. faecium has not been studied, we speculate that this might be due to the production of other unknown bacteriocins that are induced only in the absence of mutacin II. This could be a drawback of using this strain for heterologous lantibiotic screening if the peptide does not have strong activity. However, the mutacin II biosynthetic machinery is somewhat promiscuous in nature; thus, the system can be easily adapted for the production of unknown lantibiotics, especially if they belong to the lacticin 481 group. Therefore, this study has immediate biotechnological applications, at least for the food industry. Since the genome sequence is known and since the strain is easily transformable, one can collectively delete all of the putative bacteriocin loci, including sactipeptides, and *blpK* to refine the T8 strain further for use as a production/screening host.

## MATERIALS AND METHODS

### Bacterial strains and growth conditions.

Escherichia coli strain DH5α was routinely grown at 37°C in Luria-Bertani (LB) medium, and when necessary, 500 μg/mL erythromycin (Em) was added to the medium. Streptococcus mutans and other streptococci were generally grown at 37°C in Todd-Hewitt medium (BBL, BD) supplemented with 0.2% yeast extract (THY) under microaerophilic conditions. When necessary, 5 μg/mL erythromycin was included in the THY medium. All of the streptococcal strains were transformed by means of natural transformation according to standard protocols, with the addition of competence-stimulating peptides (52, 53).

### Construction of the *mutA* deletion mutant.

The structural gene of mutacin II, *mutA*, was deleted by using a fusion PCR approach as previously described (54). Briefly, ~0.5-kb upstream and downstream flanking regions were separately amplified with the primer sets T8delMutAUpF/T8delMutAKmR and T8delMutAKmF/T8delMutADnR, respectively, using the S. mutans T8 genomic DNA as a template. A Cre-LoxP-Km (kanamycin) resistance cassette was amplified from pIBM01 using primers NcoI-Kan-D7-F and PstI-Kan-D7-R (25). Next, overlapping fusion PCR was carried out with equal amounts of each PCR product using primers T8delMutAUpF and T8delMutADnR. The amplified products were purified and transformed into the S. mutans T8 strain to generate the Δ*mutA* strain, which is Km resistant. Later, the pCrePA plasmid was transformed to excise the Km cassette and finally generate a clean knockout strain, Δ*mutA*, as previously described (55).

### Construction and expression of hybrid genes.

The hybrid gene encoding the mutacin II leader peptide and the homolog core was generated by the fusion PCR method. Up- and downstream fragments were generated by producing ~500-bp PCR products that encoded 2/3 of the homolog cores both upstream and downstream, along with the homology region in the T8 chromosome. Both of the products encoded one-third of the overlap of the core region of choice. These two fragments were then used as a template in an equal ratio to generate an ~1-kb fusion PCR fragment using the flanking up- and downstream primers as mentioned above. The final PCR product was used for the transformation of the S. mutans T8 strain along with the pGh9Tr (Em resistance) plasmid in a 20:1 ratio for the markerless cotransformation method (54). Erythromycin-resistant transformants were selected and cultured in plain THY broth without the addition of any antibiotics at 37°C to cure the pGh9Tr plasmid that was used for cotransformation. The correct strains expressing only the hybrid homolog core were verified by PCR amplification of the region and sequencing.

### RT-PCR to check the expression of the hybrids.

Total RNA was extracted from S. mutans cultures as described previously (56). Briefly, cultures were freshly grown to an optical density (OD) at 600 nm of 1.0 from an inoculum grown overnight. RNA was isolated from a 10-mL culture of each strain. cDNA was prepared using Superscript-II reverse transcriptase (Invitrogen, CA). Semiquantitative RT-PCR was performed using 20 ng of cDNA for 20 cycles using the gene-specific primers listed in Table S3 in the supplemental material. The PCR product was loaded onto a 2% agarose gel, photographed, and quantitated using ImageJ. For loading and the internal control, the *gyrA* gene encoding a housekeeping gyrase was used.

### Determination of antimicrobial activity (zone of inhibition).

T8 and its mutant derivatives were stabbed onto THY agar plates and incubated under microaerophilic conditions at 37°C overnight (25). After 16 to 20 h, the plates were overlaid with cultures of the indicator strains freshly grown overnight by mixing with soft agar. When the indicator strains contained plasmids, the appropriate antibiotics were also included in the soft agar. The overlaid plates were further incubated overnight under the same conditions as the ones described above. The image of the clear zone of a halo was taken the next day. Assays were repeated at least twice, with a minimum of two replicates for each assay.
