# Supplementary material for: Synthesis of a Novel Lantibiotic Using Mutacin II Biosynthesis Apparatus
Source: Microbiol Spectr. 2023 Jan 16;11(1):e03030-22. doi: 10.1128/spectrum.03030-22 (PMC9927145; doi:10.1128/spectrum.03030-22)
Supplement: Supplemental file 1 — Supplemental material. Download spectrum.03030-22-s0001.pdf, PDF file, 1.1 MB [file spectrum.03030-22-s0001.pdf]

Table S1: Alignment of the pre-peptide mutcin II homologs

```
WP_117418566.1      1 ---------MRNDILELTNPMEEKELEQILGGG----DGVIKTISHECAMNTWQFIFTCCS
CUH82811.1          1 --------MEKFEYSNLIQEVSDSELDEILGAG----NGVIKTVSHECKMNTWQFLFTCCS
WP_002296231.1      1 -------MKQSNEMLELIQEVSLDELDQVIGGMG---KGAVGTISHECRYNSWAFLATCCS
WP_002935049.1      1 -------MKDNYELMNTIQEVSLEELDQIIGAGK---NGVFKTISHECHMNSWQFLFTCCS
WP_037580734.1      1 -------MKDNYELMNTIQEVSLEELDQIIGAGK---NGAIKTISHECHMNSWQFLFTCCS
WP_114317825.1      1 --------MKKDAVIESIKEVSLEELDQIIGAGK---NGVFKTISHECHLNTWAFLATCCS
WP_136097588.1      1 -------MKKNNEVINSIQEVSLEELDQIIGAGK---NGVFKTISHECHLNTWAFLATCCS
WP_136018286.1      1 -------MEKNNEVINSIQEVSLEGLDQIIGAGK---NGVFKTISHECHLNTWAFLATCCS
WP_175060552.1      1 -------MKKETTIIESIQEVSLEELDQIIGAGK---NGVFKTISHECHLNTWAFLATCCS
WP_093528619.1      1 -------MEKETTIIESIQEVSLEELDQIIGARK---NGVFKTISHECHLNTWAFLATCCS
WP_080004879.1      1 MNAKSNEEINYEAVATLQEVKIEDLDILLGGA----GHGVNTISAECRWNSLQAIFTCC-
WP_133363361.1      1 -------MKMNEVALTALDEVSELELNDILGAKG-KGSGVIKTLTHECKMNTYQAILTCC-
WP_074450945.1      1 --MKNTNIIDIKATEALQELSLEELDTIIGAKK--GSGVVPTVSHDCHMNSWQFLFTCCS
WP_121564619.1      1 --MKDTNIIDIEATNTLQELSLDELDTIIGAG---KEGVVPTISHDCHMNSWQFIFTCCS
WP_079260956.1      1 --MKNTNIIDIEATNALQELSFEELDTIIGAKKKGNDGAIPTISHDCHMNSWQFIFTCCS
WP_194378691.1      1 -MENLKVIEDIEVSNLLEEIQEDELNEVLGAKK--KSGAVPTVSHDCHMNSWQFIFTCCG
WP_155974693.1      1 -MENSKIMKDIEVANLLEEVQEDELNEVLGAKK--KSGVIPTVSHDCHMNTFQFMFTCCS
WP_076131461.1      1 ------MSNINMQAVSALDELSDAELDQVLG----A-EGVVTTISHECNMNSWQFIFTCCS
BDA64362.1          1 ------MSNINMQAVAALDELSDAELDEVLG----A-NGVITTISHECHLNTWAFAFTCCS
WP_076131460.1      1 ------MSNINMQAAAALDELSDAELDQVLG----AGNGVFYTFTHECNTNSFYVAFTCCS
WP_080035512.1:     1 ------MNKLNSNAVVSLNEVSDSELDTILGGNR-WWQGVVPTVSYECRMNSWQHVFTCC-
WP_074596227.1      1 ------MDKLNHSAVTSLNEVSDSELDTILGGSR-FWQGVVPTVSYECRMNSWQSIFTCC-
WP_006731084.1      1 ------MEKLNHNAVTSLNEVSDSELDTILGGSR-WWQGVLPTVSHECRMNSFQHIFTCC-
WP_080567926.1      1 ------MENLNHNAVISLNEVSDSELDTIFGGSR-WWQGVVPTVSHECNMNSFQHVFTCC-
WP_065439015.1      1 --------MNKNMTGDVLAEMTDAEFDSIIG----AGNGVVTTISHECNMNSWQFLFTCCG
WP_060391312.1      1 -------MKLNENALSSLEEVTENELDTILGA-R--KSGVINTVSHECNMNSWQFVFTCCA
EJO15235.1          1 MAKQQMNLVEIEAMNSLQELTLEELDNVLG----AGGGVIQTISHECRMNSWQFLFTCCS
ABI63639.1          1 MAKQQMNLVEIEAMNSLQELTLEELDNVLG----AGGGVIQTISHECRMNSWQFLFTCCS
WP_145517223.1      1 ------MNLVEIEAMNSLQELTLEELDNVLG----AGGGVIQTISHECRMNSWQFLFTCCS
CAA63706.1          1 ----------MTNAFQALDEVTDAELDAILG----GGSGVIPTISHECHMNSFQFVFTCCS
WP_032489363.1      1 --------MKEQNSFNLLQEVTESELDLILGA-K-GGSGVIHTISHECNMNSWQFVFTCCS
```

Table S2: Strains and plasmids used in this study

Strain or plasmid	Description	Reference or source
S. mutans:		
	T8, Wild type, serotype c	
	T8 derivative, Δ*mutA*	This study
	T8 derivative, *mutA* homolog from *Weissella cryptocercid*	This study
	T8 derivative, *mutA* homolog from *Streptococcus equi2*	This study
	T8 derivative, *mutA* homolog from *Clostridium indicum*	This study
	T8 derivative, *mutA* homolog from *Streptococcus equi1*	This study
	T8 derivative, *mutA* homolog from *Streptococcus pyogenes*	This study
	T8 derivative, *mutA* homolog from *Streptococcus suis*	This study
	T8 derivative, *mutA* homolog from *Streptococcus macedonicus*	This study
	T8 derivative, *mutA* homolog from *Streptococcus gallolyticus 1*	This study
	T8 derivative, *mutA* homolog from *Streptococcus spp.1*	This study
	T8 derivative, *mutA* homolog from *Streptococcus spp.2*	This study
	T8 derivative, mutA homolog from *Lactococcus lactis*	This Study
	T8 derivative, mutA homolog from *Staphylococcus warneri*	This Study
	T8 derivative, mutA homolog from *Streptococcus mutans 1140*	This Study
**Other strains:**		
	Acinetobacter baumanniiae B8300	Wild type
	Streptococcus anginosous 1766A	Wild type
	Streptococcus constellatus 1888B	Wild type
	Enterococcus faecium 513	Wild type
	Streptococcus agalactiae A909	Wild type
	Streptococcus gordonii DL-1	Wild type
	Streptococcus iniae K388	Wild type
	Listeria monocytogenes Gibson	Wild type
	Pseudomonas aureginosa PA14	Wild type
	Streptococcus sobrinus ATCC27352	Wild type
	Staphylococcus aureus USA300	Wild type
	Bacillus subtilis JH642	Wild type
**Plasmids:**		
pCrePA	*E. coli*-streptococcal shuttle plasmid, Em^r	

Table S3: List of oligonucleotides

Name	Sequence (5’ to 3’)	Purpose
T8delMutAUpF:	gagcatcatagtttttatcatgcccaatattttattg	*mut*A recombination
T8delMutAUpR:	cataataaaacacctcactttattgcttaaaatg	*mut*A deletion
T8delMutAKmF:	cattttaagcaataaagtgaggtgttttattatgcgataactt<br>cgtataatgtatgctatacgaagttatgaggatgaagagga	*mut*A deletion
T8delMutAKmR:	gcttaagcccccgttataatttttaatttttaaccgataactt<br>cgtatagcatacattatacgaagttatgctttttagacatctaa	*mut*A deletion
T8delMutADnF:	gttaaaaaattaaaaattataacgggggcttaagc	*mut*A deletion
T8delMutADnR:	caatcttatcagattgatctactaaaccaagattaac	*mut*A recombination
W. cryptUpR:	atcttgcactcatgagtcaaagtcttaataacaccagaa<br>cccttacccttaccacccaaaatagtatccaattcagaatc	*W. cryptocercid* recombination
W. cryptDnF:	tgactcatgagtgcaagatgaatacttatcaagcaattttga<br>cttgctgttaaaaaaattaaaaattataacggggggc	*W. cryptocercid* recombination
C. indiUpR:	agtattcattgcacactcatgagaaatagtcttaataacac<br>catcaccaccacccaaaatagtatccaattcagaatc	*C. indicum* recombination
C. indiDnF:	ttctcatgagtgtgtgttgcaatgaatacttggcaattcatttt<br>cacttgctgttcttaaaaaattaaaaattataacggggggc	*C. indicum* recombination
SgalloUpR:	gcgacactcatatgagacagttggcacaacaccttgccaga<br>aacgagaaccacccaaaatagtatccaattcagaatc	*S. gallolyticus* recombination
SgalloDnF:	tgtctcatatgagtgtcgcatgaattcatggcaatcaattttcac<br>ttgctgttaaaaaattaaaaattataacggggggc	*S. gallolyticus* recombination
SeqiUpR:	attcatatggcactcatgtgaaatagtcttaattgcaccattct<br>taccaccacccaaaatagtatccaattcagaatc	*S. equis* recombination
SeqiDnF:	cacatgagtgccatatgaattcatggcaattcttgttcacttgc<br>tgttcttaaaaaattaaaaattataacggggggc	*S. equis* recombination
SpyUpR:	gtattcaaatggcactcatgagaaatagtcttgaacaca<br>ccattcttaccaccacccaaaatagtatccaattcagaatc	*S. pyogenes* recombination
SpyDnF:	ctcatgagtgccatttgaatacttgggcattcttggcaacttg<br>ctgttcttaaaaaattaaaaattataacggggggc	*S. pyogenes* recombination
SsuisUpR:	catgaattcatatggcactcatgagaaatagtcttgaacacac<br>cattcttacc accacccaaaatagtatccaattcagaatc	*S. suis* recombination
SsuisdnF:	tatttctcatgagtgccatatgaattcatggcaattcttgttcac<br>ttgctgttcttaaaaaattaaaaattataacggggggc	*S. suis* recombination
Sequi2UpR:	ttgcaatgaattccaacggcactctgcagaaatagtat<br>tcacaccatggcctgcaccacccaaaatagtatccaattcagaatc	*S. equis*2 recombination
Sequi2DnF:	gaatactatttctgcagagtgccgttggaattcattgcaagc<br>aattttcacttgctgttaaaaaattaaaaattataacggggggc	*S. equis*2 recombination
Ssp1UpR:	gaattcatattgcactcatgagagacagttggaacaacacct<br>tgccaccaacgagaaccacccaaaatagtatccaattcagaatc	*S. spp.1* recombination
Ssp1DnF:	gttccaactgtctctcatgagtgcaatatgaattctttccaac<br>atgttttcacttgctgttaaaaaattaaaaattataacggggggc	*S. spp.1* recombination
Ssp2UpR:	catatggcaatcatgagaaatagttggaattgcaccatcattac<br>ccttcttcttaccacccaaaatagtatccaattcagaatc	*S. spp.2* recombination
Ssp2DnF:	ctatttctcatgattgccatatgaattcatggcaattcattttc	*S. spp.2* recombination

acttgctgttcttaaaaaattaaaaattataacggggggc

SmacsUpR:	gtattcaaatggcactcatgagaaatagtcttgaacacacc	*S. macedonicus* recombination
	attcttacgaccacccaaaatagtatccaattcagaatc	
SmacsDnF:	ctatttctcatgagtgccatttgaatacttgggcattcttggcaactt	*S. macedonicus* recombination
	gctgttcttaaaaaattaaaaattataacggggggc	
481UpR:	gaattcatattgcactcatgagaaatagtatgaataacacc	*L. lactis* recombination
	Agaaccacccttaccacccaaaatagtatccaattcagaatc	
481DnF:	ctatttctcatgagtgcaatatgaattcttggcaattcgttttcacttgctgttct	*L. lactis* recombination
	Taaaaaattaaaaattataacggggggc	
ISK1upR:	tgaattcatatggcaatcatgagagacagttggaataacacca	*S. warneri* recombination
	gacttcttcttaccacccaaaatagtatccaattcagaatc	
ISK1dnF:	gtctctcatgattgccatatgaattcattccaattcgttttcacttg	*S. warneri* recombination
	ctgttcttaaaaaattaaaaattataacggggggc	

sqmut2f:	cagtaacgcagtagtttctttgaatg	RT-PCR
sqcsinr:	caagtgaaaatgaattgccaagtattc	RT-PCR
sqseqi2r:	gaaaattgcttgcaatgaattccaac	RT-PCR
sqnukcnr:	tgaaaacgaattggaatgaattcatatg	RT-PCR
sqspp2r:	caagtgaaaatgaattgccatgaattc	RT-PCR
sqmut2r:	caagtgaaaacatgttgccatgaattc	RT-PCR
sq1140r:	gaattgaaagaaccagtacgtgc	RT-PCR
sqwscrr:	caagtcaaaattgcttgataagtatt	RT-PCR
sqsgalr:	gaaaattgattgccatgaattcatgc	RT-PCR
sqsmacr:	gttgccaagaatgcccaagtat	RT-PCR
sqspyor:	tgccaagaatgcccaagtattc	RT-PCR
sqssuir:	gtgaacaagaattgccatgaattc	RT-PCR
sqseq1r:	gtgaacaagaattgccatgaattc	RT-PCR
sqspp1r:	gaaaacatgttggaaagaattcatattg	RT-PCR

a.

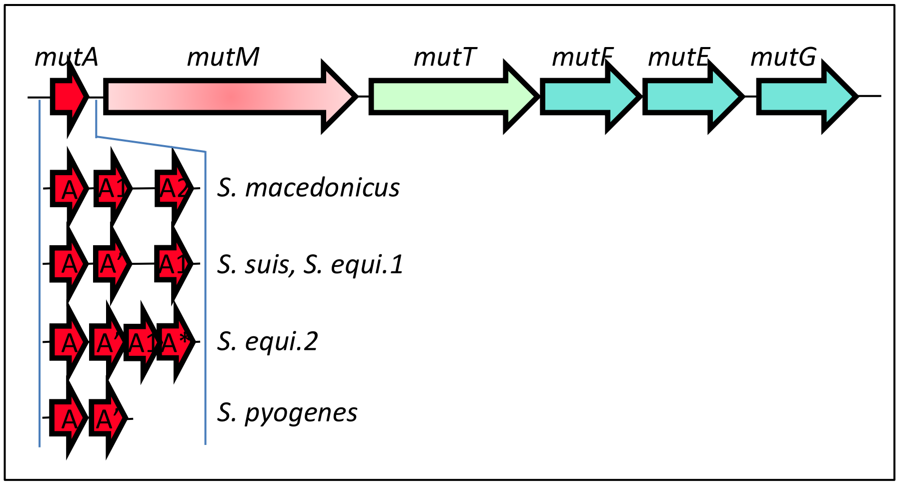

b.

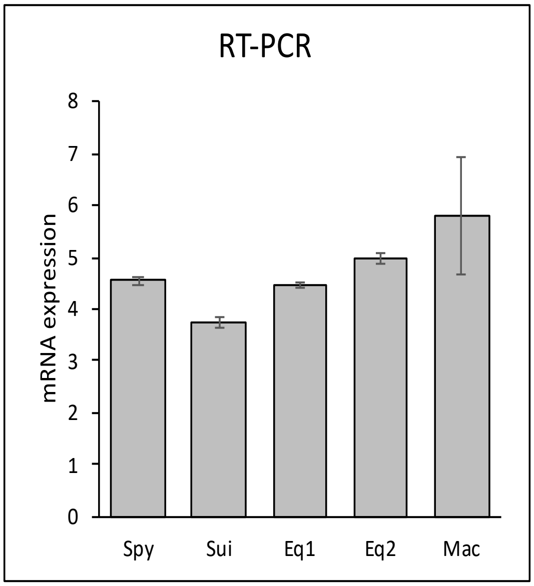

Figure S1. Evaluating expression of the homologs that are part of multipeptide clusters in *S. mutans* T8. (a) Genomic organization of the mutacin II homologs that are part of multipeptide clusters. The arrangements of the other genes vary depending on the species, shown here is the T8 locus as the base. A, A1 and A2 denote variants of one another, while A' denotes the duplication of A. A* denotes the gene that may be a pseudogene. (b) From overnight grown culture, transcriptional expression of the peptide homologs that are part of multi peptide cluster are measured by RT-PCR.

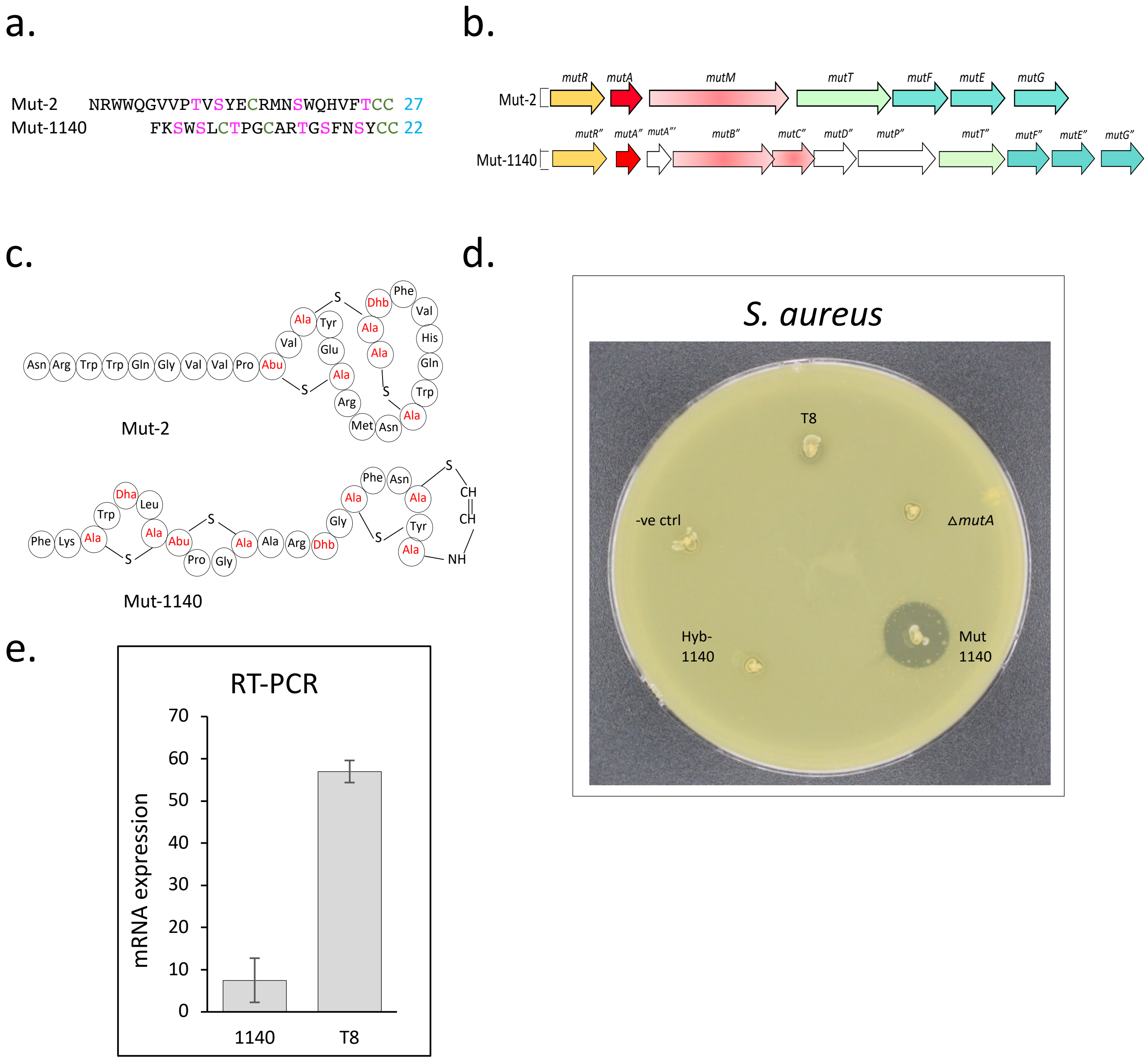

Figure S2. Evaluating expression of mutacin 1140 in *S. mutans* T8. (a) Alignment of mutacin II (Mut-2) with mutacin 1140 (Mut-1140) sequences. (b) Comparison of the biosynthetic loci of mutacin II and mutacin 1140. (c) Structure of the lantibiotics mutacin II and mutacin 1140. (d) Deferred antagonism assay of various strains. Assays were performed as described in the text and *Staphylococcus aureus* USA 300 was used as the indicator strain. The samples are: T8, wild type T8; Δ*mutA*, deletion of Δ*mutA* in T8; Mut 1140, JH1140 (mutacin 1140 producer); Hyb-1140, hybrid-1140 in T8; and -ve ctrl, nukacin Spp.2 in T8. Experiments were repeated at least two times, and a representative plate is shown. (e) Expression of the lantibiotic genes of hybrid mutacin 1140 expressed in T8 and mutacin II of T8 are measured by RT-PCR.

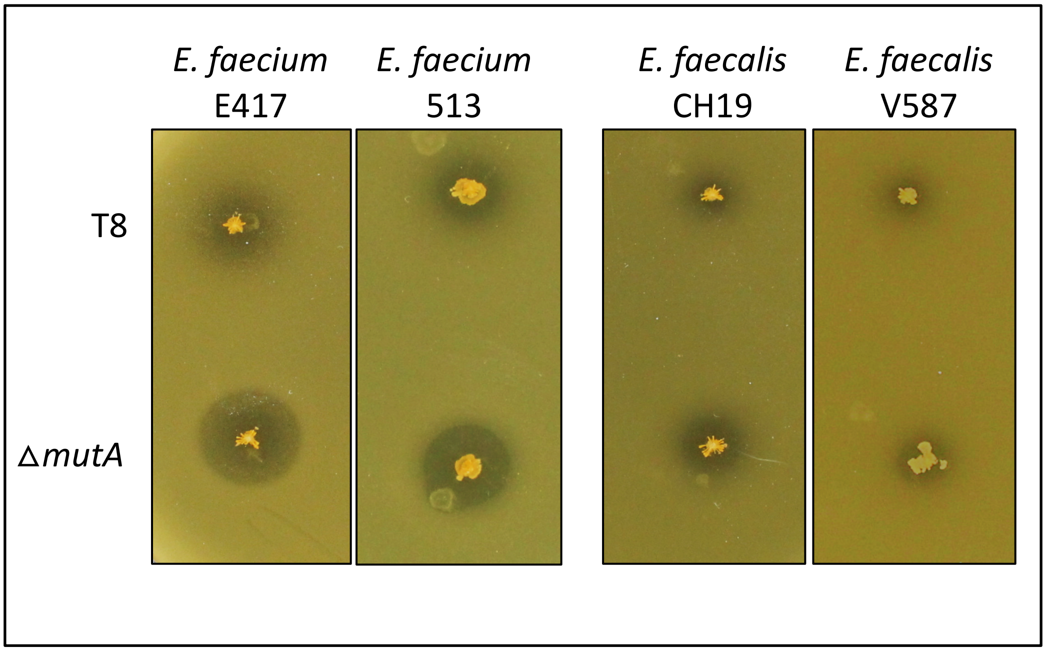

Figure S3. Halo produced by T8 is smaller than Δ*mutA* against enterococci. Halo produced by wild type T8 is smaller than that of isogenic Δ*mutA* against *Enterococcus faecium* strains E417 and 513; however, a subtle halo difference against *Enterococcus faecalis* is observed for the strain CH19 but not for the strain V587.
